# Effect of the Addition of PLA on the Thermal and Mechanical Properties of Reprocessed HDPE

**DOI:** 10.3390/polym16162387

**Published:** 2024-08-22

**Authors:** Anayansi Estrada-Monje, Miroslava Alejandra Silva-Goujon, Isis Rodríguez-Sánchez, Alain Salvador Conejo-Dávila, Claudia Ivone Piñón-Balderrama, Anayansi Zaragoza-Estrada, Leonardo Aurelio Baldenegro-Pérez, Erasto Armando Zaragoza-Contreras

**Affiliations:** 1 Centro de Innovación Aplicada en Tecnologías Competitivas, Omega No. 201, Industrial Delta, León 37545, Guanajuato, Mexico; msilva@ciatec.mx (M.A.S.-G.); alain.conejo@cimav.edu.mx (A.S.C.-D.); 2 Unidad Profesional Interdisciplinaria de Ingeniería Campus Guanajuato, Instituto Politécnico Nacional, Av. Mineral de Valenciana No. 200, Col. Fraccionamiento Industrial Puerto Interior, Silao de la Victoria 36275, Guanajuato, Mexico; isrodriguez@ipn.mx; 3 Centro de Investigación en Materiales Avanzados, S.C. Miguel de Cervantes No. 120, Complejo Industrial Chihuahua, Chihuahua 31136, Chihuahua, Mexico; claudia.pinon@cimav.edu.mx (C.I.P.-B.); 180175-8@iberoleon.edu.mx (A.Z.-E.); armando.zaragoza@cimav.edu.mx (E.A.Z.-C.); 4 Centro de Ingeniería y Desarrollo Industrial, Av. Playa Pie de la Cuesta No. 702, Desarrollo San Pablo, Santiago de Querétaro 76125, Querétaro, Mexico; leonardo.baldenegro@cidesi.edu.mx

**Keywords:** biodegradable polymer, high-density polyethylene, poly(lactic acid), polymer blend, recycling, thermal properties

## Abstract

Amid the current environmental crisis caused by plastic accumulation, one of the proposed solutions to manage this problem is using biodegradable polymers. However, the impact of adding biodegradable polymers to the well-established circular economy of recyclable polymers, such as HDPE, has not been fully considered. Therefore, there is a need to reconsider the way we consume, dispose of, and manage biodegradable polymers after use. This study evaluates the effect of varying the contents of a biodegradable polymer, taking poly(lactic acid) (PLA) as a model biodegradable polymer, on the thermal and mechanical properties of HDPE. The study highlights the importance of identifying and disposing of biodegradable polymers to avoid mixtures with HDPE, in order not to affect mechanical performance when considering reprocessing and a new life cycle of this conventional polymer.

## 1. Introduction

According to the last reports, 390 million tons of plastic were produced worldwide in 2021. Among those plastics, polyolefins are the most abundant, including polyethylene, available in applications like bags, toys, pipes, and packaging goods. In this context, 353 million metric tons of plastic waste were created, according to a new report from the Organization for Economic Cooperation and Development (OECD) [1]. To overcome this problem, actions have been taken to promote plastic recycling. Thus, plastics like poly(vinyl chloride) (PVC), low-density polyethylene (LDPE), and high-density polyethylene (HDPE) with 22% recycling are the most important. However, contamination with other polymers during the recycling process is an obstacle for recycling. This issue may limit the reutilization of recycled HDPE (rHDPE) in some applications [2]. Along with the tendency to increase recycling, using biodegradable polymers to decrease plastic waste contamination has also increased. According to this, the rise in popularity of biodegradable plastics, eco-friendly materials that do not harm the environment, as an alternative to traditional non-biodegradable petroleum-based polymers highlights the need to investigate the impact of biodegradable polymers on waste management. These biodegradable materials are not specifically labeled and are often disposed of in the same manner as conventional polymers, resulting in unknown mixtures during reprocessing.

For example, high-density polyethylene (HDPE), a commonly used petroleum-based polymer, is collected at a rate of 26% in Mexico, and its recycling rate is steadily increasing [1]. Therefore, it is crucial to study the effects on HDPE properties when blended with a biodegradable polymer at the end of its lifecycle to continue increasing the percentage of HDPE that is recycled without affecting its performance properties.

Studies have focused on enhancing the resistance of poly(lactic acid) (PLA) by blending it with polyolefins [2]. However, the lack of chemical interactions between the components often leads to an incompatible blend. As a result, compatibilization is necessary to improve adhesion between the phases. However, using compatibilizing agents may impact the PLA’s hydrolysis resistance and, consequently, its biodegradability.

In this context, there have been numerous investigations to solve concerns of incompatibility between polyolefins and PLA [2,3,4,5] and improved PLA mechanical properties. Nevertheless, three important aspects, (a) the PLA biodegradability, (b) the impact of the PLA incorporation on a recycled polyolefin, and (c) the effect of varying amounts of PLA on the well-established circular economy of HDPE, have not been considered.

The present study aims to evaluate the impact of incorporating poly(lactic acid) (PLA) into recycled high-density polyethylene (HDPE) and the effect of poor separation techniques on the thermal properties of the blends towards the end of their lifespan, as well as after a second lifespan following the recycling. Specifically, the thermal transitions of HDPE blended with PLA, with varying weight contents of 0, 10, 30, and 50%, were studied after two re-processing cycles. The research provides a comprehensive overview of the recycling process, particularly as pertains to replacing conventional plastics with biodegradable materials. By exploring the behavior of these materials in various scenarios, this study aims to gain insight into their suitability for sustainable industrial practices. The novelty of this work derives from its investigation of mechanical and structural properties in terms of PLA content in blends with HDPE.

## 2. Materials and Methods

### 2.1. Materials

High-density polyethylene (HDPE 65050, PEMEX, supplier in León, Gto.) and poly(lactic acid) (PLA 3251D, Nature Works, Plymouth, MN, USA) were used as delivered. PLA has a melting temperature (T_m_) of 155–170 °C, glass transition temperature (T_g_) of 55–60 °C, density of 1.240 g/cm^3^, and melt flow rate of 80 g/10 min (210 °C/2.16 kg). HDPE has a melt flow rate of 4.0–6.0 g/10 min (190 °C/2.16 kg), density of 0.9635–0.9695 g/cm^3^, and T_m_ of 130 °C.

### 2.2. Recycling Method

To obtain the recycled materials, virgin HDPE was processed using an injection molding machine (MTH-55, Milacron, Cincinnati, OH, USA), with a temperature profile from 190 to 210 °C as reported in Table 1. The material was ground and the process was repeated to obtain C1 (material recycled once). PLA was dehydrated on a stove for 6 h at 80 °C. Then it was blended with HDPE. The blends were processed twice to obtain C2. The processing cycle consists of injection, cooling, reduction of particle size by blade milling, and injection to obtain test pieces.

### 2.3. Characteristics of Samples

The samples were characterized using a tension machine (Universal testing machine, Instron, Norwood, MA, USA) provided with a 5 kN load cell, according to the ASTM D-638 [6] procedure. The tensile strength was determined at a rate of 50 mm/min. Five testing pieces were prepared from each blend. In addition, impact strength was assessed using a low-energy plastic impact tester (IT503, Tinius Olsen, San Diego, CA, USA), according to the ASTM D-256 [7] method. The notched samples were tested with a 4J pendulum.

Chemical characterization of recycled polymer (C1 and C2) and the polymer blends was carried out using an infrared spectrometer (Nicolet iS10, Thermo Fisher Scientific, Waltham, MA, USA) on the 4000–400 cm^−1^ interval with 16 scans per sample by the method of attenuated total reflectance (ATR). A detailed profile of the oxidation index (OI), trans-vinylene index (VI), and crystallinity index (CI) was obtained, as a function of the number of recycled cycles (0, 1, and 2). The spectra were used for the calculation of the oxidation index (OI, Equation (1)), trans-vinylene index (VI, Equation (2)), and crystallinity index (CI, Equation (3)):

(1) $$\mathrm{OI}=\frac{A_{1720}}{A_{1370}}$$

(2) $$\mathrm{VI}=\frac{A_{965}}{A_{1370}}$$

(3) $$\mathrm{CI}=\frac{A_{1897}/A_{1303}}{A_{1897}/A_{1303}+0.3}$$

The details of OI, VI, and CI determination can be consulted in the literature [8,9,10]. In the CI index, the infrared absorption at 1897 cm^−1^ is assigned to the crystalline phase, whereas the one at 1303 cm^−1^ is assigned to the amorphous phase in HDPE according to Slouf Miroslave et al. [11].

Thermal characterization was performed using a differential scanning calorimeter (DSC Pyris 1, PerkinElmer, Waltham, MA, USA). Samples of 8 mg were used. Assays were run with a heating rate of 15 °C/min up to 250 °C, under a nitrogen atmosphere, and the temperature was held for 30 s (first heating scan) before cooling. Then, the system was cooled to 40 °C at the same rate (second scan). The glass-transition temperature (T_g_), melting temperature (T_m_), crystallization enthalpy (ΔH_c_), and melting enthalpy (ΔH_m_) were registered during the second heating scan. The crystallinity degree was calculated from the melting enthalpy and the enthalpy of 100% crystalline HDPE, taken as 288 J/g [12]. The percent crystallinity of the blends was calculated according to Equation (4) [3].

(4) $$\%\;\mathrm{crystallinity}=\left(\frac{{∆H}_{c}/\varphi_{\mathrm{HDPE}}}{{∆H}^{0}}\right)\times 100$$

where φ_HDPE_ is the HDPE content in the blend, ΔH_c_ is the melting enthalpy of the blend, and ΔH^0^ is the heat of fusion of 100% crystalline HDPE (293 J/g) [13].

## 3. Results

**Figure 1:**
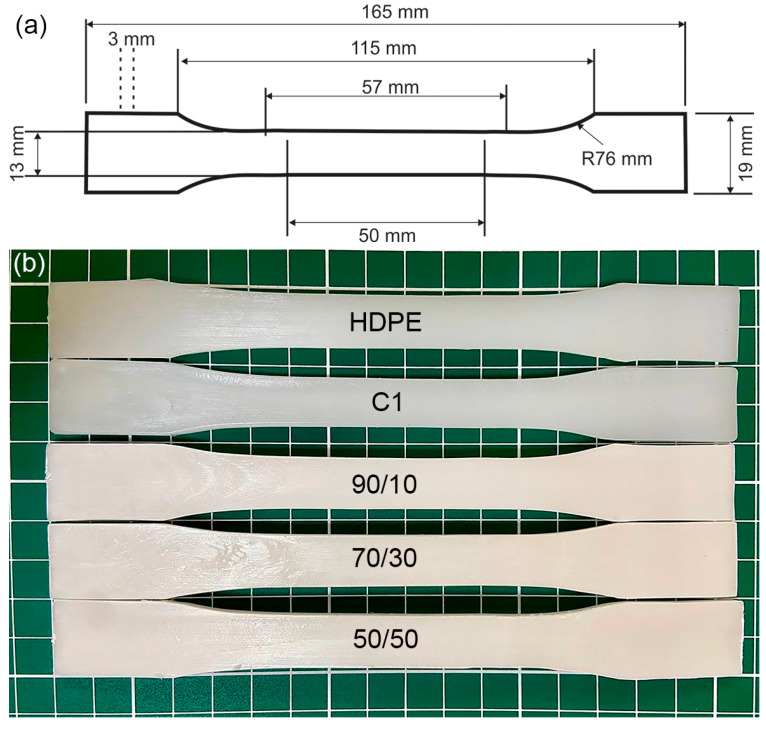
(**a**) Specifications of ASTM D 638 type 1 standard and (**b**) mechanical test specimens (from top to bottom), virgin HDPE, C1, HDPE/PLA 90/10, HDPE/PLA 70/30, HDPE/PLA 50/50.

The samples were injected and type 1 samples of ASTM D 638 standard were obtained. Figure 1 shows the probes of pure HDPE, C1, and HDPE/PLA blends. A slight color change can be observed when comparing the HDPE probe with C1 (HDPE with one recycling cycle). Furthermore, once HDPE is blended with PLA, the sample appears slightly whiter as the PLA content increases. It should be noted that after reprocessing, the samples become slightly yellow even with only one recycling cycle.

### 3.1. Mechanical Properties

**Figure 2:**
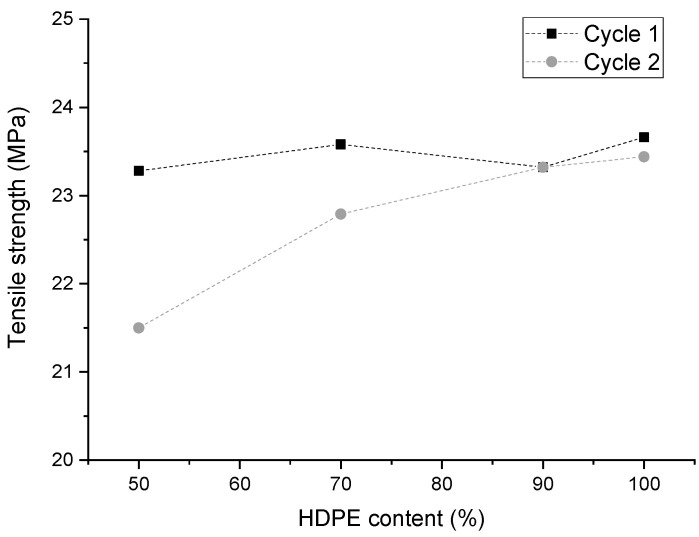
Tensile strength of the blends varying the HDPE/PLA content ratio.

To complement characterization, the blends’ tensile strength was evaluated. Figure 2 exhibits a change in the tensile strength of the blends that undertook one and two recycling cycles, varying the amount of PLA in the blend.

As observed, with the first recycling cycle, the tensile strength remains constant with a small variation when the PLA content varies. With the second recycling process, the tensile strength decreases as the PLA content increases, from 23.44 to 21.50 MPa. As reported in the literature, the tensile strength of HDPE/PLA blends decreases gradually as the amount of PLA increases. This continues until the tensile strength shows a drastic decrease when the PLA content reaches 20% [14], indicating a progressive increase in brittleness. However, we found that the tensile strength was maintained in the case of one recycling cycle, and slightly increased with the second recycling cycle. As pointed out previously, this augmentation is thought to be linked to the increase of HDPE crystallinity, promoted by the byproducts of PLA degradation as the number of recycling cycles increases. Another indication of PLA degradation is the standard deviation of the tensile strength, which increases with the number of recycling cycles (Table 2).

### 3.2. Functional Group Analysis

**Figure 3:**
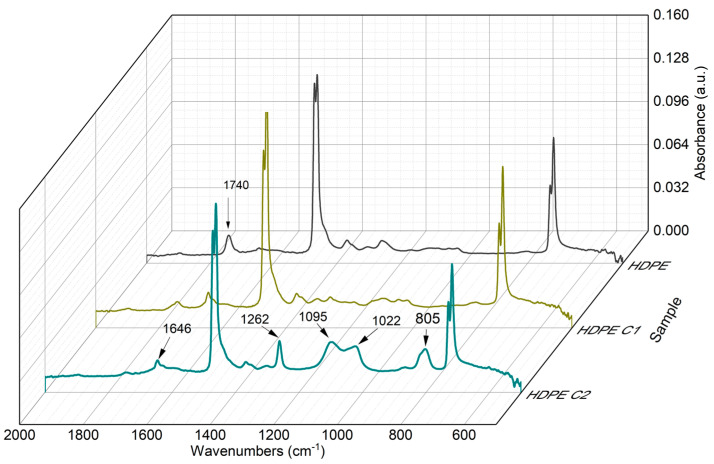
Infrared spectra in the 600 to 2000 cm^−1^ interval of virgin HDPE, HDPE C1, and HDPE C2.

FTIR spectroscopy was used to determine the chemical changes of the HDPE surface during recycling, providing a clear view of the material’s degradation. Figure 3 shows the FTIR spectra of virgin HDPE and HDPE with one and two recycling cycles.

Spectra display the characteristic absorptions of polyethylene at 2850 and 2912 cm^−1^ assigned to CH stretching in e –CH_2_- groups (not included), at 1470 cm^−1^ to the bending band of –C–H bonds of –CH_2_ groups, and at 722 cm^−1^ to the rocking mode of –CH_2_ of the sequence of methylene groups in the alkyl structure [15]. It is worth noting the notable differences in the spectra of the virgin material, compared to those after one or two recycling cycles. The spectrum section from 600 to 2000 cm^−1^ is analyzed to highlight the differences.

Figure 3 compares the spectra of the virgin HDPE, C1, and C2. The range from 2000 to 4000 cm^−1^ is not included, but absorptions of polyethylene at 2850 and 2912 cm^−1^ ascribed to CH stretching in –CH_2_ groups are present [16]. The absorption at 1646 cm^−1^, assigned to the formation of unsaturations and terminal vinyl groups, the peak at 1262 cm^−1^, ascribed to the asymmetric C–CH bending, and the band at 805 cm^−1^, related to an increase in the formation of trisubstituted vinyl groups of the R–CH=CH–R’ type, as well as the increase of the trans-vinylene groups’ index, are according to Equations (6) and (7). On the other hand, a difference in the band at 1740 cm^−1^, corresponding to the formation of ketones due to the oxidation of HDPE [14], is observed while comparing the spectra of virgin HDPE, C1, and C2, possibly due to the diverse reactions between peroxides and alkyl radicals to form carbonyl groups.

The absorption bands near 1260–900 cm^−1^ are associated with the vibrational mode of C–O stretching [17]. In this region (Figure 3), specific differences between the HDPE and the recycled materials were found. The differences were attributed to oxidation reactions in the polymer chain. Zang et al. [18] also noted the presence of carbonyl groups as a distinctive indicator of HDPE degradation. These authors employed bacteria for HDPE degradation; however, we consider that the effect of shear and temperature can generate the same oxidized species.

(5) $$\mathrm{RH}+O_{2}\overset{\mathrm{yields}}{\to }R^{.}+{\mathrm{HOO}}^{.}$$

(6) $$R-{\mathrm{CH}}_{2}{\mathrm{CH}}_{2}\dot{\mathrm{CH}-}R_{1}\overset{\mathrm{yields}}{\to }\;R-\dot{{\mathrm{CH}}_{2}\;}+{\mathrm{CH}}_{2}=\mathrm{CH}-R_{1}$$

(7) $$R-\dot{{\mathrm{CH}}_{2}}+R-\dot{{\mathrm{CH}}_{2}}\overset{\mathrm{yields}}{\to }R-\mathrm{CH}={\mathrm{CH}}_{2}+{\mathrm{CH}}_{3}-R_{1}$$

**Figure 4:**
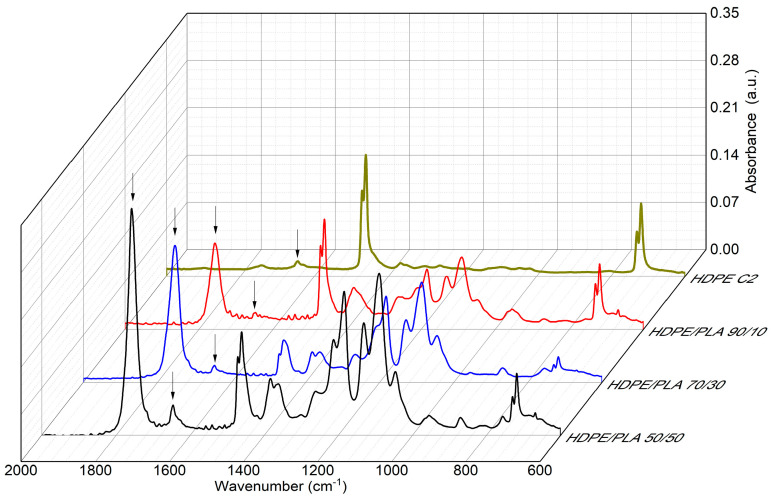
Infrared spectra in the 600 to 2000 cm^−1^ interval of the blends of HDPE/PLA with two recycling cycles. The arrows indicate the differences among the absorption bands in the infrared spectra.

Figure 4 shows the spectra of HDPE and HDPE/PLA blends at concentrations of 90/10, 70/30, and 50/50. In all the blends, the absorption bands corresponding to HDPE are visible, as previously discussed; the absorption bands corresponding to PLA are also visible, such as the bands in the 1780–1680 cm^−1^ region assigned to the stretching of the C=O group, the bands at 1750–1180 cm^−1^ assigned to the stretching of C–O–C, and the band at 1454 cm^−1^ to the stretching of CH_3_, among others. As reported in the literature, the oxidation of PLA can be established by observing the change in the carbonyl band [19]; in the spectrum of the blends, it is observed that the intensity of the carbonyl band increases as the concentration of PLA in the blend increases, as expected. However, a slight shift in the carbonyl peak towards longer wavelengths was also observed compared to virgin PLA which is found at 1750 cm ^−1^ in the blends, as the absorption band shifts up to 1758 cm^−1^. Similar observations were made by Badia et al. when they performed multiple reprocessings on PLA; this shift was explained as an indication of the formation of new species attached to the carbonyl and as an indication of a reduction in molar mass in the material [19,20]. On the other hand, as observed in the figure, the band at 1646 cm^−1^ in HDPE, assigned to the unsaturations and terminal vinyl groups that increase with reprocessing, also presents a significant increase with increasing PLA concentration in the blend, and this finding could indicate that the degradation of PLA in reprocessing favors the increase of species with unsaturation in HDPE.

Table 3 reports the detailed profiles corresponding to OI, VI, and CI, as a function of the number of recycling cycles (0, 1, and 2).

As observed in Table 3, an increase in the OI of HDPE occurred when comparing the virgin materials with the ones processed once and twice. In addition, an increase in VI is also present, which is consistent with the differences in the absorptions at 1646, 868, and 803 cm^−1^ (Figure 3).

The reprocessing of HDPE seems to have a minimal effect on the crystallinity, as will be seen later for the thermal transitions analyzed by DSC. However, a CI was obtained from the infrared spectrum, where the absorptions at 1897 cm^−1^ and 1303 cm^−1^ are assigned to the crystalline and amorphous phase in HDPE [10]. As noted for CI, a slight decrease in the materials’ crystallinity is observed as the number of reprocessings increases, from 0.65 in virgin HDPE to 0.59 in C2. This is consistent with the percentage crystallinity calculated from DSC, which will be discussed later. Czarnecka-Komorowska et al. [21] reported a crystallinity increment in reprocessed HDPE blends (aged by UV light). These authors reported HDPE recrystallization due to the heat generated, which was related to the degraded molecules’ recrystallization and crosslinking after UV irradiation. However, in this work, an increase of crystallinity was not observed, but rather a slight decrease, likely because two recycling cycles are insufficient to cause a critical molecular degradation, which facilitates the polymeric chains’ reorganization.

**Figure 5:**
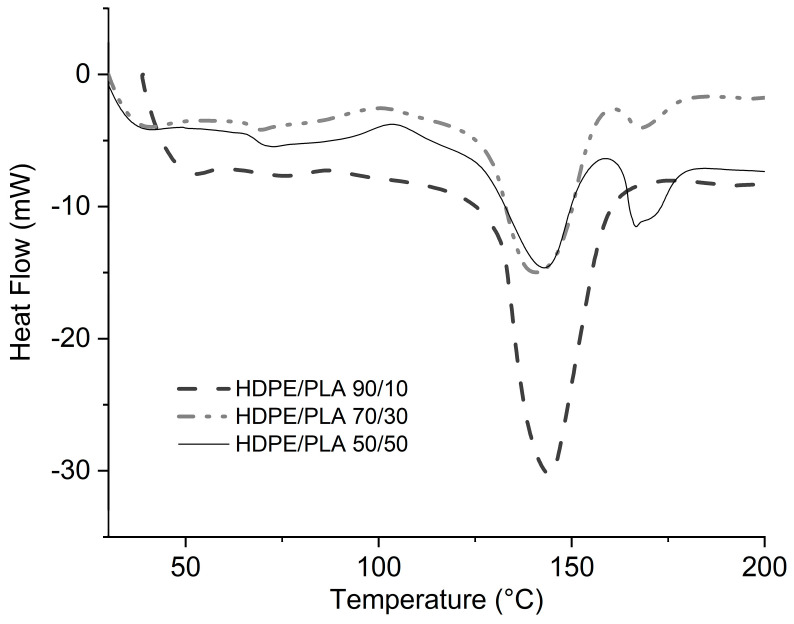
DSC curves of the HDPE/PLA blends.

Figure 5 depicts the DSC thermograms of the three HDPE/PLA blends. Exothermic transitions around 140 °C and 170 °C, corresponding to the HDPE crystalline melting and the crystalline melting of PLA, respectively, are present. The intensity variation of both transitions depends on the PLA content, as expected, due to the variation in crystalline phase content and the effect that PLA has on the crystallization process of HDPE.

### 3.3. HDPE/PLA Blend Analysis

Table 4 lists the values of crystallinity and OI of the HDPE/PLA blends submitted to two reprocessing cycles. Crystallinity, crystallinity percentage, and χ were obtained from FTIR and DSC (Equation (2)).

As observed, adding 10 wt% PLA causes a decrease in the HDPE crystallinity in both reprocessing cycles. As pointed out in the previous section, the reprocessing of pure HDPE does not appear to affect crystallinity; consequently, this decrease seems to result from PLA presence. The literature indicates that PLA presence affects the formation of critical nuclei for the spherulite growth, the chain diffusion across the polymeric matrix to the growing spherulites, and the mass center diffusion of the crystallizable phase of HDPE to the growth front [14,22]. PLA also affects the HDPE chain mobility, which hinders the chain rearrangement and thus influences the crystallization process. However, a higher amount of PLA seems to favor the HDPE crystallization process, as noted by Quitadamo et al. [4], who reported an increase in crystallinity of 50:50 HDPE/PLA blends and an improvement in mechanical properties. Seemingly, PLA promotes the crystallization processes, as the increment in blend crystallinity is caused by the interactions of the compounds generated from the thermo-oxidative degradation of PLA during the reprocessing cycles.

According to the literature, PLA accelerates the degradation of HDPE by the carboxylated chain formation during PLA degradation [14]. Generally, PLA reduces the negative environmental impact of plastic waste, accelerating the HDPE degradation, and modifying the crystallinity while incorporating PLA without using compatibilizing agents.

## 4. Conclusions

The recycling of plastics is a crucial strategy for minimizing waste and repurposing materials. HDPE recycling is a critical process for plastic recovery, making it essential to investigate any alterations to its composition that may impact its properties. As environmental regulations become more stringent, biodegradable plastics are becoming more prevalent. However, it remains unclear how they will interact with conventional plastics once they reach the waste stream. One potential outcome is that the biodegradable polymer’s breakdown could be impeded by unsuitable conditions. Recycled plastics, such as HDPE, may also experience mechanical property changes. Consequently, blends of HDPE with PLA are not miscible and, in such a two-phase blend, the morphology and thus the physical properties are controlled by the weight ratio of the two components and the interfacial adhesion between the phases. Poor interfacial compatibility of the polymer components leads to separation between the phases at low applied stress, followed by crack formation and embrittlement. All this happens if an appropriate coupling agent is not used. It is worth mentioning that in this study no coupling agent was used, to simulate what would happen if the materials were taken from the trash and attempted to be recycled; obviously, all the other components that may be present in polymers to be recycled, such as dirt, were not considered.

This study demonstrated that varying amounts of PLA affect HDPE’s mechanical properties and that the effect increases as the reprocessing cycles of the material increase. Therefore, the crystallization of HDPE is affected by the presence of PLA. This modification influences the blend’s mechanical behavior, as evidenced by the tensile strength tests. It is important to note that as the number of recycling cycles grows, the blend’s tensile strength will eventually decrease, while both polymers’ thermo-oxidative degradation increases, leading to an adverse failure of the material.

## Figures and Tables

**Table 1:** Temperature profile for the injection process of HDPE/PLA blends.

Blend (wt%)	Temperature (°C)		
HDPE/PLA	Zone 1	Zone 2	Zone 3
100/0	190	200	210
90/10	190	200	210
70/30	190	200	210
50/50	190	200	210
0/100	190	200	210
HDPE C1 *	190	200	210
HDPE C2 **	190	200	210

* HDPE C1 recycled once, ** HDPE C2 recycled twice.

**Table 2:** Tensile strength and standard deviation.

Material	Tensile Strength (MPa)
Virgin PLA	22.41 ± 1.20
Virgin HDPE	23.69 ± 0.65
HDPE C1	23.66 ± 0.67
HDPE C2	23.44 ± 0.66
Cycle 1	
HDPE/PLA 90/10	23.38 ± 0.64
HDPE/PLA 70/30	23.58 ± 0.85
HDPE/PLA 50/50	23.28 ± 0.85
Cycle 2	
HDPE/PLA 90/10	23.32 ± 1.16
HDPE/PLA 70/30	22.79 ± 0.67
HDPE/PLA 50/50	21.50 ± 1.21

**Table 3:** Trans-vinylene index (VI), crystallization index (CI), crystallinity (χ), oxidation index (OI), and melting temperature (T_m_).

Material	CI	χ (%)	OI	VI (×10^3^)	T_m_
HDPE	0.67	57.8	0.001	2.77	132
C1	0.65	57.6	0.38	3.60	135
C2	0.59	57.4	0.90	4.46	135

**Table 4:** CI, χ, and OI of HDPE/PLA blends.

Material	Cycle 1			Cycle 2		
	CI	χ (%)	OI	CI	χ (%)	OI
HDPE	0.65	58.6	0.38	0.59	58.4	0.90
HDPE/PLA 90/10	0.22	43.9	3.8	0.98	42.2	16.4
HDPE/PLA 70/30	0.94	68.5	5.9	0.98	52.4	18.9
HDPE/PLA 50/50	0.98	61.6	7.6	0.96	57.1	25.4

## Data Availability

Data are contained within the article.
